# Accurate Prediction of Central Conventional Chondrosarcoma Risk and Outcome Using Methylation Profiling: CHROME

**DOI:** 10.1002/gcc.70160

**Published:** 2026-08-04

**Authors:** Sara Cardoso, Baptiste Ameline, Sanne Venneker, Debora M. Meijer, Zeynep B. Erdem, Dina Ruano, Brendy E. van den Akker, Anne‐Marie Cleton‐Jansen, Jon Brugger, Inge H. Briaire‐de Bruijn, Claire H. J. Scholte, Michiel A. J. van de Sande, Felix Haglund de Flon, Daniel Baumhoer, Noel F. C. C. de Miranda, Judith V. M. G. Bovée

**Affiliations:** ^1^ Department of Pathology Leiden University Medical Center Leiden the Netherlands; ^2^ Leiden Center for Computational Oncology Leiden University Medical Center Leiden the Netherlands; ^3^ Bone Tumor Reference Center at the Institute of Pathology University Hospital Basel and University Basel Basel Switzerland; ^4^ Department of Orthopedic Surgery Leiden University Medical Center Leiden the Netherlands; ^5^ Department of Clinical Pathology and Cancer Diagnostics Karolinska University Hospital Stockholm Sweden; ^6^ Basel Research Centre for Child Health Basel Switzerland; ^7^ Department of Pathology Netherlands Cancer Institute Amsterdam the Netherlands

**Keywords:** bone tumor, chondrosarcoma, CpG island, DNA methylation, isocitrate dehydrogenase, risk score

## Abstract

Chondrosarcomas are malignant cartilage‐forming bone tumors with heterogeneous behavior, making prognostication and clinical management challenging. Histological grading is the primary tool for predicting clinical outcomes in conventional chondrosarcoma. However, its high interobserver variability limits reliable distinction between low‐ and high‐risk patients, potentially leading to suboptimal clinical management. Given the increasing use of DNA methylation profiling as a valuable tool in surgical pathology for tumor classification, we investigated its prognostic value in central conventional chondrosarcoma. We generated methylation data from 69 primary central conventional chondrosarcomas profiled with Illumina's *Human MethylationEPIC Array* (850 k sites), and identified methylation sites individually linked to patient outcome. A LASSO Cox regression model was applied to these methylation sites to identify an optimal set of eight informative sites. With the regression coefficients of these methylation sites, we constructed a risk score that predicts central conventional Chondrosarcoma Risk Outcome from Methylation (CHROME). CHROME stratifies patients into *High* or *Low risk* groups for disease recurrence, onset of metastasis and disease‐specific mortality. Survival analysis in an independent validation cohort (*n* = 68) demonstrated strong discriminatory performance of CHROME, with complete separation of outcomes and no adverse events observed in the *Low risk* group. Overview of the clinico‐pathological information showed that a low grade (ACT/G1) case with an event was correctly assigned to the *High risk* group, while nine high grade cases without events were classified as *Low risk*. CHROME provides accurate risk stratification in central conventional chondrosarcoma and may represent a valuable tool for improving its prognostication and clinical management.

## 1 Introduction

Central conventional chondrosarcoma is a malignant cartilage‐producing tumor that arises in the medullary cavity of bone. It can affect any part of the skeleton formed by endochondral ossification, including long tubular bones, flat bones, and occasionally the spine or skull base. This tumor predominantly affects adults aged 30–60 years, with an equal sex distribution [1, 2].

Histological grading is the most important predictor of clinical outcome guiding clinical management of chondrosarcoma. Central atypical cartilaginous tumors/chondrosarcoma grade 1 (ACT/G1) have a 5‐year overall survival (OS) rate of 87%–99%, with local recurrences in 7.5%–10% of cases. ACT is by definition located in the appendicular skeleton and is either followed up with repeated MRI or treated with curettage and local adjuvants, while G1 chondrosarcoma—same morphology but located in the axial skeleton—requires surgical excision with negative margins [1]. Grade 2 (G2) and grade 3 (G3) chondrosarcomas require more extensive, en bloc resection and wider negative margins, with 5‐year OS rates of 58%–86% and 26%–55%, respectively. Local recurrences occur in 19% of G2 and 26% of G3 cases, with metastases in 10%–30% and 32%–71%, respectively [2, 3]. The ability to reliably distinguish low‐ and high‐risk lesions is crucial to prevent both over‐ and under‐ treatment.

Histological grading is subject to high interobserver variability. A study showed that the concordance rate among 9 pathologists grading 46 cases was low (kappa (*k*) coefficient of 0.443). In a subgroup of outcome‐determined high‐risk patients (10 cases), reliability decreased further (*k* = 0.236), with grades ranging from low‐ to high‐grade, and in some cases from benign to high‐grade [4]. In another study of 57 cases, the concordance was slightly better (*k* = 0.78) [5].

Several attempts have been made to find better prognostic biomarkers in chondrosarcoma, including the mutational status of the isocitrate dehydrogenase (IDH) 1 and 2 genes. Heterozygous hotspot mutations in *IDH1* and *IDH2*, which lead to DNA hypermethylation, are present in about 50% of central chondrosarcomas and 87% of benign cartilage tumors, indicating a significant role in their development [6, 7, 8, 9, 10]. The prognostic value of IDH mutations remains unclear. While some studies report no effect on outcomes [6, 7, 8, 11], Lugowska et al. [9] found worse OS for IDH‐mutant cases with no impact on recurrence‐free survival. Conversely, Zhu et al. [10] reported no effect on overall, metastasis‐free, or relapse‐free survival but found better metastasis‐ and relapse‐free survival for high‐grade (G2 and G3) IDH mutant cases.

Given the increasing use of DNA methylation profiling in surgical pathology for classification of tumors [12, 13, 14, 15], we investigated its prognostic value for patients with chondrosarcoma.

As a result, we developed Chondrosarcoma Risk Outcome from Methylation (CHROME), an objective methylation‐based risk score that accurately stratifies patients into low‐ and high‐risk groups for disease‐free survival (DFS).

## 2 Methods

### 2.1 Discovery Cohort

Fresh‐frozen, treatment naïve, tissue from the primary tumor of 69 patients diagnosed with central conventional chondrosarcoma was retrieved from the LUMC bone and soft tissue tumor biobank (Leiden, the Netherlands) to construct the discovery cohort. Approval of institutional review board (Medisch‐Ethische Toetsingscommissie Leiden Den Haag Delft) was obtained for the use of the tissue samples (B20.067). Written informed consent from patients was obtained. All specimens were pseudo‐anonymized and handled according to the ethical guidelines described in “Code for Proper Secondary Use of Human Tissue in The Netherlands” of the Dutch Federation of Medical Scientific Societies. The diagnosis, including the histological grade, was reviewed and confirmed by two pathologists (J.V.M.G.B. and Z.B.E.).

DNA was isolated from fresh‐frozen tissue using the Wizard Genomic DNA purification kit (A1125, Promega, Madison, WI, USA), according to the manufacturer's instructions. The IDH mutation status was assessed using the Cancer hotspot panel (CHPv6, Department of Pathology, Leiden University Medical Centre, https://www.palga.nl/media/uploads/pdf/4/9/496_102‐chpv6‐lumc.pdf [16]), performed by GenomeScan (Leiden, the Netherlands) as described previously [17]. The IDH mutation hotspots were *IDH1* p.R132 or *IDH2* p.R172. DNA CpG methylation profiling was carried out using Illumina's *Human MethylationEPIC Array* (850 k) platform, version 1.0, according to the manufacturer's instructions.

Clinical and follow up data were obtained from clinical files. Follow‐up time was calculated from date of surgery to the date of first event (local recurrence, metastasis or death from any cause) or date of last follow‐up for DFS.

### 2.2 Validation Cohort

We collected two datasets independent of the discovery cohort to construct the validation cohort. Only central chondrosarcoma cases that fulfilled the following criteria were selected: (1) raw DNA methylation data (IDAT files) from the primary tumor available, obtained using an Illumina Methylation array with no customization; (2) follow‐up information regarding at least deaths; (3) clear information on grade and, if possible, IDH mutation status, age, and sex. About 54 cases were thus collected from Nicolle et al. [11] (*Remy* dataset), and 14 other cases from the Karolinska Institutet in Stockholm (*Karolinska* dataset) [18]. The *Remy* dataset comprised Illumina's *Infinium HumanMethylation450 K* data while the Karolinska dataset was generated with Illumina's *MethylationEPIC array* (930 K) platform, version 2. In the Karolinska dataset, 3 samples corresponded to fresh‐frozen tissue while 11 samples were derived from formalin‐fixed paraffin‐embedded tissues. The Remy dataset comprised only fresh‐frozen tissue samples.

Follow‐up time was calculated similarly to the discovery cohort.

### 2.3 Data Processing

The R package *minfi* (v1.44.0) [19, 20, 21] was used to process all the samples. Raw signal intensities were obtained from the IDAT files. Discovery and validation cohorts were checked for quality control individually.

Samples were corrected for the background and dye‐bias. The multidimensional scaling (MDS) plot (R package *limma* (v3.54.0) [22]) revealed sex as the main source of variation in the data. Probes targeting the X and Y chromosomes were thus filtered. Low‐quality probes (detection *p* value > 0.01), probes known to have common SNPs at the CpG site, and probes not mapping uniquely to the genome were also excluded.

Raw intensity values were transformed into *M*‐values for statistical analysis.

### 2.4 Construction of the Methylation‐Based Risk Function

Using the discovery cohort, we constructed a methylation‐based risk function to classify patients into *Low* and *High risk* using DFS.

#### 2.4.1 Clustering and Differential Methylation Analysis

Samples were grouped according to graph‐based clustering, using the R package *bluster* (v1.8.0) [23]. Clustering was performed with a shared nearest‐neighbor (SNN) graph of 10 nearest neighbors, whose communities were detected using the *Louvain* clustering method. Several clustering resolutions were tested, and the best one (1.5) was chosen with the help of a clustergram, using the R package *clustree* (v.0.5.1) [24].

Differential methylation analysis between the groups was performed by R package *limma* (v.3.54.0) [22]. Methylation sites were considered differentially methylated if the absolute fold change (log_2_) was higher than 2 and the corrected *p* value (Benjamini and Hochberg [25]) was smaller than 0.01.

#### 2.4.2 Univariable Cox Proportional Hazards Analysis

Survival analysis was performed for each differentially methylated site using univariable Cox proportional hazards analysis to assess associations with patient outcome. The methylation status of a specific site had a significant effect on patient outcome if the corrected *p* value (Benjamini and Hochberg [25]) was smaller than 0.05 and the confidence interval did not include the hazard ratio (HR) 1. The R packages *survival* (v3.4‐0) [26] and *contsurvplot* (v0.2.0) [27] were used.

#### 2.4.3 LASSO Cox Regression and the Methylation‐Based Risk Function

Methylation sites individually associated with patient outcome were further evaluated to identify a minimum subset of sites that, together, were strongly associated with patient survival and captured nonoverlapping survival‐related variability. LASSO Cox regression was applied to identify such subset using a lasso penalty function to optimize the sites' regression coefficients. This penalty function shrinks the coefficients toward zero and depends on the value of the parameter *λ*. The optimal value of *λ* was determined by applying the LASSO Cox regression with a 10‐fold cross‐validation. The R package *glmnet* (v4.1‐7) [28, 29] was used.

Only those with a final coefficient other than 0 are part of the final subset of methylation sites that were used to construct the methylation‐based risk function. Since negative coefficients indicate a positive effect in outcome, the higher the risk score, the higher the risk of poor outcome. The risk function was constructed in the following manner:

$$\sum_{i=1}^{N}{\text{coef}}_{\text{site}\_i}*M{\text{value}}_{\text{site}\_i}$$

where *N* is the number of sites with a coefficient other than zero. For each site *i*, the LASSO's coefficient is multiplied by the *M*‐value of the site.

#### 2.4.4 From a Numeric to a Categorical Risk Score

Using the mean of the risk scores of the discovery cohort's samples, we established a cut‐off value of −0.9 in order to separate samples into *High* or *Low risk*.

#### 2.4.5 Alternative Methylation‐Based Risk Function

Since one of the datasets from the validation cohort included data generated with the Illumina platform *Human Methylation450 K*, which does not include two methylation sites used in the risk score function, we adapted the function by using two surrogate methylation sites. Using the discovery dataset (*MethylationEPIC v1.0* array), we calculated the correlation between those two methylation sites and the remaining sites that are present in both the *MethylationEPIC v1.0* and *Methylation450 K* arrays. After this, those two sites were substituted by the one with which they correlated the most. The sites' coefficients, as well as the cut‐off value between *High* and *Low risk* samples, were maintained.

#### 2.4.6 Validation of the Methylation‐Based Risk Function

The alternative and original methylation‐based risk functions were applied to the validation cohort to independently assess how well the risk score predicts outcome of central chondrosarcoma samples not used in the construction of this function. Samples were separated into *High* and *Low risk* scores based on the cut‐off value established in the discovery cohort (−0.9).

#### 2.4.7 Influence of Clinical Features in Risk Score

The influence of histological grade, IDH mutation status, age and sex in the risk score was assessed in the validation cohort. First, univariable Cox proportional hazards analysis was performed for each of these features alone. Only those with an individual significant effect on survival were tested together with the risk score using multivariable Cox proportional hazards analysis. Because Cox proportional hazards analysis works best on continuous variables instead of categorical, categorical features were converted into numerical values. IDH mutation status and sex were binarized. For histological grade, the higher the grade, the higher the numerical value: (1) ACT/G1, (2) G2, (3) G3. The R packages *survival* (v3.4‐0) [26] and *contsurvplot* (v0.2.0) [27] were used.

### 2.5 Kaplan–Meier Survival Estimates

Kaplan–Meier survival estimate was performed for several categorical variables in this study. Log‐Rank test was used to assess if the difference between the respective categories was significant. The Kaplan–Meier method and log‐rank test were performed using the R packages *survival* (v3.4‐0) [26] and *survminer* (v0.4.9) [30].

### 2.6 Data Visualization

Samples were visualized in the 2‐dimensional space using the Uniform Manifold Approximation and Projection (UMAP) algorithm. UMAPs were performed on the 10 000 most variable methylation sites, using the R package *uwot* (v0.1.14) [31]. Heatmaps were constructed using the R packages *ComplexHeatmap* (v2.14.0) [32], *circlize* (v0.4.15) [33] and *grid* (v4.2.2). R package *ggpubr* (v0.5.0) [34] was used to arrange different plots together.

## 3 Results

### 3.1 Methylation Profiles Associate With Disease‐Free Survival in Chondrosarcoma

To construct a methylation‐based risk score, we performed DNA methylation profiling of 69 chondrosarcomas using Illumina's Human MethylationEPIC v1.0 array. Patient characteristics, including histological grading, are provided in Table 1.

**TABLE 1:** Characteristics of patient samples included in the discovery and validation cohorts.

Characteristics			Validation (*n* = 68)	
		Discovery (*n* = 69)	*Remy* dataset [11] (*n* = 54)	*Karolinska* dataset (*n* = 14)
Grade	ACT/G1	ACT = 20 (28.99%)	ACT = 7 (12.96%) G1 = 1 (1.85%)	G1 = 1 (7.14%)
	G2	30 (43.48%)	32 (59.26%)	5 (35.71%)
	G3	19 (27.54%)	14 (25.93%)	8 (57.14%)
IDH status	MUT	43 (62.32%)	31 (57.41%)	5 (35.71%)
	WT	26 (37.68%)	19 (35.19%)	8 (57.14%)
	*NA*	0	4 (7.41%)	1 (7.14%)
TERT promoter status	MUT	5 (7.25%)	NA	NA
	WT	64 (92.75%)	NA	NA
Age	Mean ± standard deviation (years)	52.14 ± 13.83	53.61 ± 17.24	56.64 ± 9.76
Sex	Female	32 (46.38%)	17 (31.48%)	7 (50%)
	Male	37 (53.62%)	37 (68.52%)	7 (50%)
Location	Head and Neck	1 (1.45%)	2 (3.70%)	1 (7.14%)
	Foot and Hand	0	4 (7.41%)	0
	Vertebra	2 (2.90%)	2 (3.70%)	2 (14.29%)
	Flat Bones	29 (42.03%)	26 (48.15%)	6 (42.86%)
	Long Bones	37 (53.62%)	20 (37.04%)	5 (35.71%)
Maximum tumor size	Mean ± standard deviation (cm)	9.30 ± 7.88	NA	NA
Follow‐up	Local Recurrences	11 (15.94%)	NA	2 (14.29%)
	Metastases	7 (10.14%)	NA	4 (28.57%)
	Deaths	5 (7.25%)	13 (24.07%)	5 (35.71%)
	Disease (total)	15 (21.74%)	*NA*	5 (35.71%)
	Time to Last Follow‐up (months)	0.8 to 288.62 (mean: 83.72)	0.45 to 178 (mean: 58.09)	4.68 to 307.07 (mean: 127.01)
Tissue	Fresh Frozen	69 (100%)	54 (100%)	3 (21.43%)
	FFPE	0	0	11 (78.57%)
Array type		*HumanMethylationEPIC v1.0*	*HumanMethylation450 K*	*HumanMethylationEPIC v2.0*

*Note:* Grade: ACT/G1—central atypical cartilaginous tumor/chondrosarcoma grade 1; G2—chondrosarcoma grade 2; G3—chondrosarcoma grade 3. IDH Status: mutation status of isocitrate dehydrogenase 1/2, containing either one of the known (IDH1 R132 or IDH2 R172) hotspot mutations (MUT), or not (WT) or information not available (NA). Age is represented by the mean, with respective standard deviation.

**FIGURE 1:**
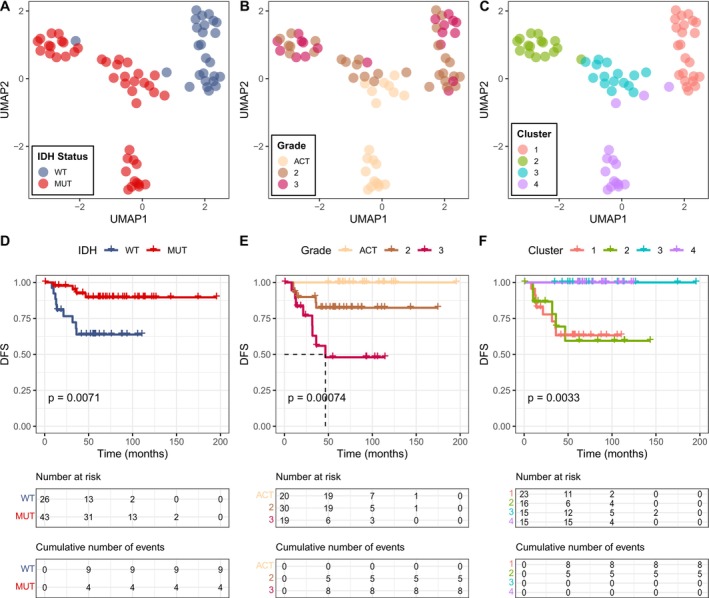
Visualization of discovery cohort's cases in the two‐dimensional space using UMAP and Kaplan–Meier survival estimate curves of disease‐free survival (DFS). Plots are colored according to (A) IDH mutational status, (B) histological grade, (C) graph‐based clusters. Kaplan–Meier Survival estimate curves of DFS for (D) IDH mutational status, (E) histological grade, and (F) graph‐based clusters, with respective log‐rank test's *p* value. *MUT*: IDH hotspot mutation present; *WT*: IDH hotspot mutation absent. *Number at risk*: number of cases through time with no event or not censored yet. *Cumulative number of events*: cumulative number of cases through time with an event.

Visualization of sample separation based on methylation profiles using UMAP showed a clear distinction between IDH mutated and IDH wild‐type cases (Figure 1A). Regarding histological grading, ACT/G1 cases tended to separate from grade 2 and grade 3 chondrosarcomas (Figure 1B).

As anticipated, Kaplan–Meier survival estimates showed a significant difference in DFS between histological grades (Figure 1E), with grade 3 having the worst performance and ACT/G1 the best. IDH mutation status was also significantly associated with DFS, with IDH‐mutated cases showing better survival compared to wild‐type cases (Figure 1D). This effect appeared to be primarily driven by the fact that all ACT/G1s in our dataset were IDH‐mutated (Figure S1). Anatomical location was also significantly associated with DFS (Figure S2A), although primarily driven by the ACT/G1s occurring in long bones (Figure S2B). TERT promoter mutational status (Figure S2C) and tumor size (HR 0.98, *p* value = 0.794) showed no significant association with DFS.

Clustering of samples according to their methylation profiles revealed four distinct clusters (Figure 1C, Figure S3) with the following predominant pathological and molecular features: (Cluster 1) IDH wild‐type, grades 2 or 3; (Cluster 2) IDH mutant, grades 2 or 3; (Cluster 3) IDH mutant, ACT/G1 or grade 2; and (Cluster 4) IDH mutant, ACT/G1. Anatomical location showed relation with IDH mutational status (Figure S4A), as reported previously [35], with most cases localizing to flat or long bones. Tumor size and TERT promoter mutational status showed no particular association with any cluster (Figure S4B,C). Clusters 1 and 2 exhibited worse DFS compared to clusters 3 and 4, which had no events (Figure 1F). Strikingly, some cases in cluster 3 corresponded to grade 2 or 3 chondrosarcomas.

### 3.2 CHROME: A Methylation‐Based Risk Score Composed of Eight Methylation Sites That Predicts DFS in Chondrosarcoma

To construct a methylation‐based risk score, we began by assessing differentially methylated sites between clusters (1) + (2) and clusters (3) + (4).

Out of the total 803 324 sites in the discovery dataset, 14 597 were differentially methylated (corrected *p* value < 0.01, |log fold‐change| > 2, Table S1). Of these, 12 049 displayed increased methylation in clusters (3) and (4), while only 2548 sites displayed increased methylation in clusters (1) and (2).

Next, we performed survival analysis on each differentially methylated site to determine their individual effect on survival. This was done using univariable Cox proportional hazards analysis for each methylation site separately.

From the 14 597 sites examined, 9274 were individually associated with DFS (corrected *p* value < 0.05, HR not equal to 1 within the 95% confidence interval, Table S1). Among these, 7719 were positively associated with survival, that is, higher methylation on these sites associated with better DFS, while 1555 had a negative relation.

To avoid overfitting the risk score to the discovery cohort and to eliminate highly correlated methylation sites, we applied a LASSO Cox regression model to define an optimal set of informative sites. After using a LASSO penalty function to optimize each site's regression coefficient and selecting those with a final coefficient other than zero, we identified 8 suitable methylation sites (Table 2).

**TABLE 2:** Genomic annotation of the methylation sites used in CHROME, with respective risk coefficient.

CHROME						
Site	Gene	Chr	Band	Annotation	Strand	Risk coefficient
cg09678323^a^	DNM3	1	q24.3	Intron	+	−0.01348094
cg05391318	NR5A2	1	q32.1	Intron	+	0.04522507
cg06597895	TGM4	3	p21.31	Exon	+	−0.08337541
cg08030922	SNCA	4	q22.1	Intron	−	−0.24067993
cg23242862	LOC401463	8	q12.3	Intron	−	0.11434405
cg00253658		16	q12.2	Intergenic	−	0.17099035
cg07323648	LHX1‐DT	17	q12	Intron	−	0.08220902
cg06031622^b^	MANBAL	20	q11.23	Intron	+	−0.03142363
450 K‐compatible CHROME						
cg10663897^a^		2	q24.2	Intergenic	+	−0.03142363
cg25336892^b^		2	q23.1	Intergenic	−	−0.01348094

*Note:* A negative risk coefficient translates into higher methylation related with better disease‐free survival, while a positive one is related with worse disease‐free survival. Methylation sites highlighted with ^a^ or ^b^ are those that were substituted in the *450 K‐compatible* CHROME.

The final risk score can be calculated based on the LASSO's coefficients and the methylation sites' *M*‐values: − (0.03142363*cg06031622) − (0.24067993*cg08030922) − (0.01348094*cg09678323) + (0.11434405*cg23242862) + (0.08220902*cg07323648) − (0.08337541*cg06597895) + (0.04522507*cg05391318) + (0.17099035*cg00253658). Since negative coefficients indicate a positive association with outcome, a higher risk score correlates with a higher risk of developing local recurrences, metastasis and death.

Thereafter, a score was calculated for each chondrosarcoma case. Samples were further separated into *High* and *Low risk* groups based on a cut‐off of −0.9, defined using the mean of the cases' risk values of the discovery cohort. Thus, samples with a methylation‐based risk score higher than −0.9 are classified as *High risk*, while those with scores lower than −0.9 are classified as *Low risk*. This risk score is called CHROME, for its role in predicting central conventional CHondrosarcoma Risk Outcome from MEthylation.

**FIGURE 2:**
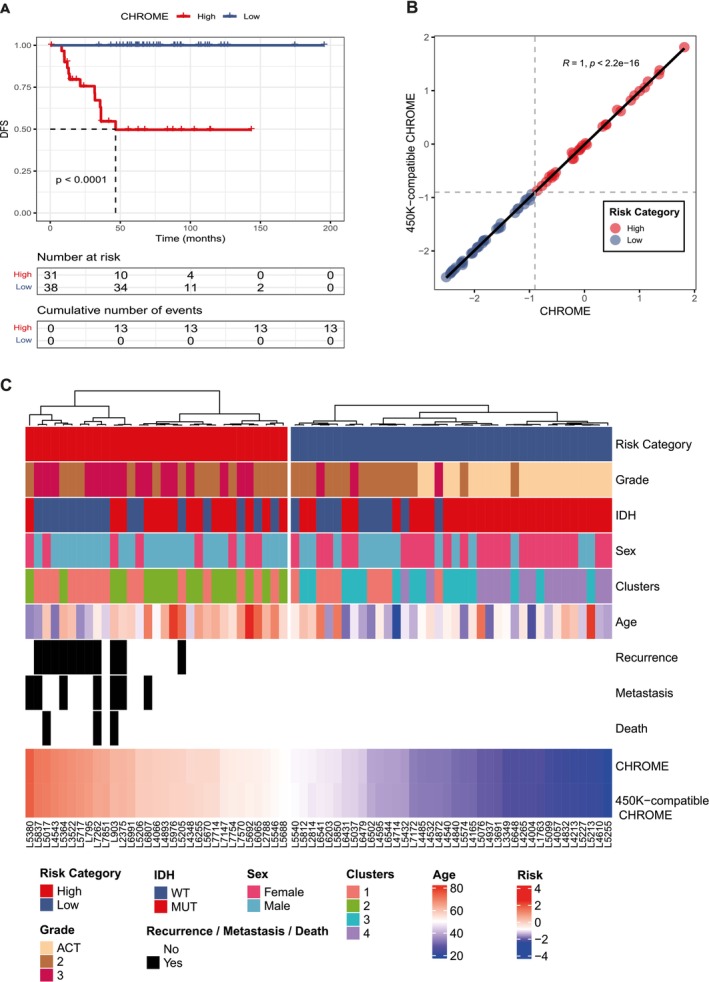
CHROME results in the discovery cohort. (A) Kaplan–Meier survival estimate curves of disease‐free survival (DFS) for categorical CHROME risk. (B) Correlation between the original risk score (CHROME) and the risk score adapted for 450 K Illumina arrays (450 K‐compatible CHROME). (C) Overview of clinical information and risk scores of cases. *MUT*: IDH mutated; *WT*: IDH wild‐type. *CHROME*: original risk score. *450 K‐compatible CHROME*: risk score adapted to be compatible with Illumina's *Human Methylation450 K* arrays. *Number at risk*: number of cases through time with no event and not censored yet. *Cumulative number of events*: cumulative number of cases through time with an event.

The Kaplan–Meier survival estimates of DFS for the categorical risk showed a significant difference between the *High risk* (*n* = 31) and *Low risk* (*n* = 38) groups, with all 13 events occurring in the *High risk* group (Figure 2A,C). This significance remained when looking into the grades 2 and 3 cases alone (Figure S5), with no shorter follow‐ups in the *Low risk* group (Table S2). High‐grade cases classified as *Low risk* (*n* = 18) showed characteristic histomorphological features of high‐grade cases (Figure S6).

Although the differentially methylated sites between the graph‐based clusters (1) + (2) and (3) + (4) served as a starting point for constructing the risk score, the *High risk* / *Low risk* characterization of the cases was not a direct result of the clusters. From the 39 cases in the poor outcome clusters (1) and (2), 24 had no events. From these, 8 were classified as *Low risk* by CHROME (Figure 2C).

### 3.3 Validation of CHROME's Prognostic Value

To validate CHROME, we applied it to an independent validation cohort of 68 chondrosarcomas (Table 1). About 54 cases were collected from Nicolle et al. [11] (*Remy* dataset), and 14 other cases from the Karolinska Institutet in Stockholm (*Karolinska* dataset) [18]. While all samples in *Remy* dataset were derived from fresh‐frozen tissue, only 3 of the 14 samples from the *Karolinska* dataset corresponded to fresh‐frozen tissue. The remaining 11 samples were derived from formalin‐fixed paraffin‐embedded tissues.

The *Remy* dataset was generated using an Illumina methylation array different from that used in the discovery cohort. Because the *Human Methylation450 K* array only covers approximately 450 000 CpG sites and did not include two CHROME methylation sites, we adapted the model by identifying, in the discovery cohort, the 450 K sites most highly correlated with these two sites. The two missing methylation sites were replaced accordingly (Figure S7), and the resulting score is hereafter referred to as the 450 K‐compatible CHROME risk score. In the discovery cohort, CHROME and 450 K‐compatible CHROME risk scores highly correlated with each other (Figure 2B), with no case changing risk category. Furthermore, for the validation cases where the original CHROME score could be applied, there was a good correlation between CHROME and 450 K‐compatible CHROME scores, with no changes on risk category (Figure S8).

**FIGURE 3:**
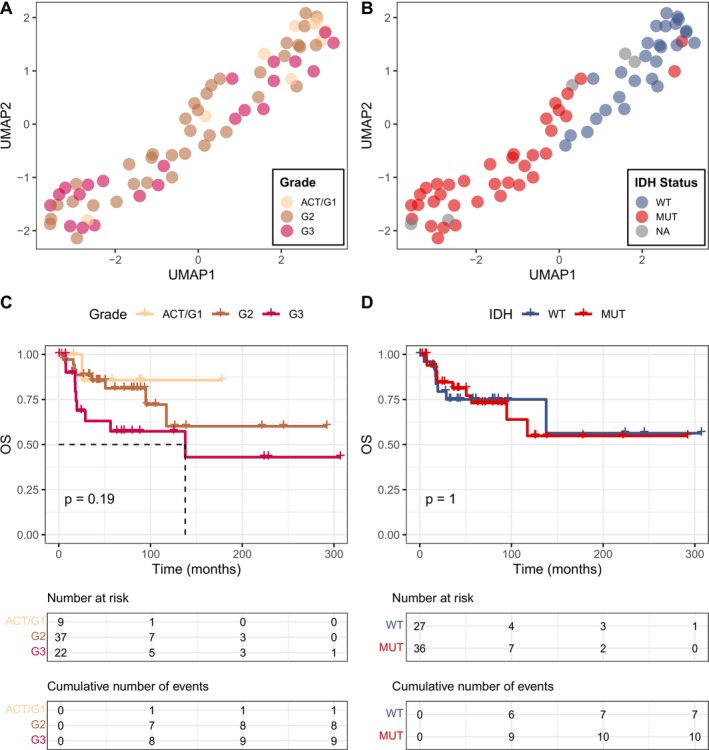
Visualization of validation cohort's cases in the 2‐dimensional space using UMAP and Kaplan–Meier survival estimate curves of overall survival (OS). UMAP Plots are colored according to (A) histological grade and (B) IDH mutational status. Kaplan–Meier survival estimate curves of OS for (C) histological grade and (D) IDH mutational status, with respective log‐rank test's *p* value. *MUT*: IDH mutated; *WT*: IDH wild‐type. *Number at risk*: number of cases through time with no event or not censored yet. *Cumulative number of events*: cumulative number of cases through time with an event.

We inspected the methylation profile of the validation cohort cases in the 2‐dimensional space using the UMAP algorithm (Figure S9). Contrary to the discovery cohort (Figure 1), there were only two groups of cases, with no ACT/G1 group separate from the rest (Figure 3A). There was a separation, however, between IDH mutated and IDH wild‐type cases (Figure 3B), just like with the discovery cohort (Figure 1A). Anatomical location showed no particular grouping (Figure S10).

**FIGURE 4:**
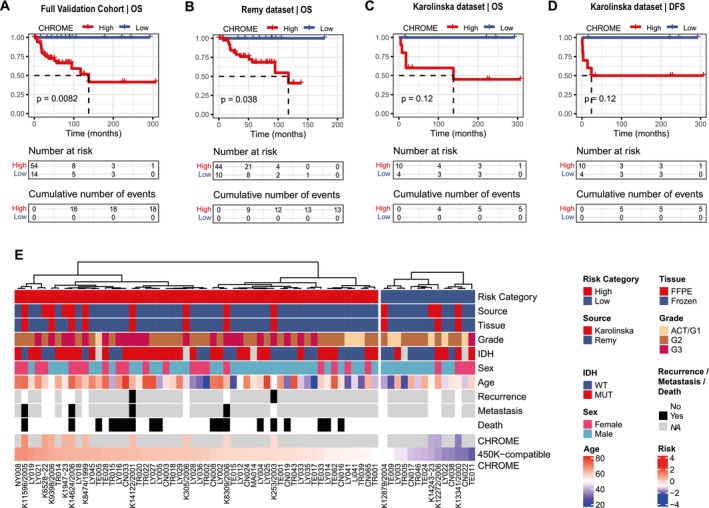
Validation of the CHROME risk score. Kaplan–Meier survival estimate curves of overall survival (OS) for categorical CHROME risk for (A) the full validation cohort, (B) only the Remy dataset, and (C) only the Karolinska dataset. (D) Kaplan–Meier survival estimate curves of disease‐free survival (DFS) for the Karolinska dataset. (E) Overview of clinico‐pathological information and risk scores of cases.

One of the datasets in the validation cohort only provided information regarding deaths from any cause (Table 1). Thus, CHROME was firstly validated for OS in the whole validation cohort. Of note, histological grade, IDH mutational status and anatomical location showed no significant effect on OS (Figure 3C,D and Figure S11). Patients classified as *High risk* (*n* = 54) by 450 K‐compatible CHROME showed significantly worse survival than the *Low risk* (*n* = 14) samples (Figure 4A). This behavior was similar in both datasets that constituted the validation cohort (Figure 4B,C).

The *Karolinska* dataset provided further follow‐up information regarding local recurrences and metastasis (Table 1). Thus, despite the relatively low number of cases (*n* = 14), we assessed the performance of CHROME on this dataset for DFS. Even though not significant, events only occurred in *High risk* samples (Figure 4D), with a similar result for only the FFPE samples (Figure S12).

Similar results for CHROME's risk score were obtained when looking into the grades 2 and 3 cases alone (Figure S13), with no shorter follow‐ups observed in the *Low risk* group (Table S2).

Overview of the clinico‐pathological information and risk scores of the validation cases (Figure 4E) showed that not all low grade (ACT/G1) cases were classified as *Low risk*, as well as not all high‐grade (grades 2 and 3) cases were classified as *High risk*. The low grade case with an event was correctly classified as *High risk*, while 9 *high grade* cases (grade 2: *n* = 8, grade 3: *n* = 1) without an event were classified as *Low risk*.

We further assessed the effect of clinicopathological features and numerical risk score on OS for the validation cohort, using univariable Cox proportional hazards analysis. Both 450 K‐compatible CHROME and age showed a significant effect on OS (Table S3). Multivariable analysis was then conducted on these two variables in the validation cohort, and none showed significance, likely due to the low number of events.

## 4 Discussion

Predicting the clinical behavior of conventional central chondrosarcoma on histological specimens is difficult as histological grading is observer dependent and no prognostic biomarkers have made it into clinical application so far. In this study, we have developed CHROME, a score that predicts central conventional CHondrosarcoma Risk Outcome from MEthylation. This score uses the methylation status of only 8 sites in the genome that can be derived from methylation arrays to confidently categorize primary chondrosarcoma samples into *High* or *Low risk* of developing local recurrences, metastasis, and death. CHROME's prognostic value was confirmed in an independent validation cohort.

CHROME score can be derived from different methylation platforms. One of the datasets used in the validation cohort was generated using an Illumina platform (*Human Methylation450 K*) different than the one used in the discovery cohort (Illumina's *Human MethylationEPIC v1.0*), with two CHROME methylation sites not present in the older platform. This limitation led to the creation of a second version of CHROME that could be applied to the older methylation array. Results showed that these two versions are highly correlated, allowing CHROME to be reliably derived from two different versions of methylation arrays. In the future, CHROME can be adapted and validated to new versions in the same way it was adapted for the *Human Methylation450 K* array. Furthermore, CHROME could be applied to both samples derived from fresh‐frozen and FFPE tissue, demonstrating its applicability in distinct diagnostic contexts.

Even though CHROME was developed based on DFS, it was shown to also be prognostic for OS, given the lack of DFS data in one of the validation cohort's datasets (*Remy dataset*). CHROME was capable of classifying all samples from patients with death events as *High risk*, thus effectively separating *Low* from *High risk* groups. Nevertheless, it should be noted that it is uncertain whether all death events in the *Remy dataset* were related to disease.

The *Karolinska dataset* in the validation cohort had further information regarding local recurrences and metastasis, making it possible to test CHROME on this subset of cases for DFS, the same clinical endpoint used to train CHROME. Even though the number of samples was not large enough to reach significance, all cases with events were classified as *High risk*.

Although inherent to the rarity of the disease, the limited number of events present in both discovery and validation cohorts constitutes a limitation of the study. This creates a need to further validate the findings, including the cutoff value between *Low* and *High risk* groups, in a bigger cohort with the same clinical endpoint as the discovery cohort, DFS. Nevertheless, CHROME shows promising potential to be used as a prognostic tool for conventional central chondrosarcoma.

So far, histological grade has been the best predictor of outcome in conventional chondrosarcoma, despite several attempts to find superior prognostic markers. Additional prognostic markers have been proposed [36, 37], though most of them were not independent of histological grade and none of them have been implemented in clinical practice. Multiple studies have investigated the prognostic value of IDH mutations with opposing results [6, 7, 8, 9, 10, 11]. In the discovery cohort of the current study, the IDH mutation was associated with improved disease‐free survival, while such an association was absent in the validation cohort. The effect in the discovery cohort seemed to be caused mainly by all ACT/G1s in the cohort being IDH‐mutated, suggesting that discrepant results in the literature could be explained by the heterogeneity of the different cohorts.

In addition to IDH mutational status, histological grade was also significantly associated with DFS in the discovery cohort, as expected. Surprisingly, no significant association with OS was observed in the validation cohort, although high‐grade cases showed a trend toward poorer prognosis. This may be explained by the underrepresentation of low‐grade (ACT/G1) tumors, which accounted for only 9 out of the 68 validation cases. In the discovery cohort, a subset of ACT/G1 cases exhibited low tumor cell percentage and high bone marrow content [18]. These cases were retained to ensure that CHROME was trained under conditions reflecting a real‐world clinical setting, where such samples may occur. Although these event‐free cases were consistently ranked among the lowest risk tumors, some cases with low bone marrow content also received similarly low risk scores. Importantly, all cases with events were classified as *High risk* in both the discovery and validation cohorts, including one low‐grade case in the validation cohort. Conversely, a subset of high‐grade cases without events was consistently classified as *Low risk*.

The genes related to the methylation sites that compose CHROME provide valuable insights on the biological processes that might be affecting outcome in central conventional chondrosarcoma. These processes include driving the production of the important cartilaginous matrix components aggrecan and type II collagen (*TGM4*) [38], cytoskeleton remodeling and cell motility (*SCNA*, *DNM3*) [39, 40, 41, 42], autophagy (*SCNA*) [39], cytokinesis and regulation of cell cycle (*NR5A2*) [43, 44, 45], and promotion of anti‐ or pro‐ inflammatory immune responses (*NR5A2*, *LOC401463*) [46, 47, 48].

Methylation profiling is increasingly used in surgical pathology for classification of tumors [12, 13, 14, 15]. While in brain tumors its diagnostic validity has been established [12], methylation profiling for the classification of mesenchymal tumors has great potential but requires further improvement [14, 15, 49, 50]. Classification of conventional chondrosarcomas has shown satisfactory results, with 59.42% of cases being correctly classified as chondrosarcomas, 37.68% unclassified, and only 2.90% incorrectly classified [49]. In a separate study, our discovery cohort was extended including also enchondromas, clear cell and dedifferentiated chondrosarcomas, to address the diagnostic use of methylation patterns [18]. Unsupervised dimensionality reduction of genome‐wide DNA methylation patterns revealed four clusters among IDH mutant tumors including one cluster predominantly, but not exclusively, containing enchondromas and ACT/G1 chondrosarcomas. The great potential of using methylation profiling as a routine diagnostic tool in sarcoma opens the door to easily integrate CHROME into practice to improve prediction of outcome in tumors diagnosed as central conventional chondrosarcoma, after follow‐up studies with a higher number of cases are conducted. Other more cost‐effective alternatives could be tested, like a methylation‐specific multiplex ligation‐dependent probe amplification (MS‐MLPA) test, or even a methylation specific PCR, designed to specifically analyze the methylation status of CHROME's eight sites. Advantages of using MS‐MLPA include its low cost and small amounts of DNA required.

In conclusion, we developed CHROME, a clinically meaningful methylation‐based risk score for central conventional chondrosarcoma, compatible with Illumina's Human MethylationEPIC and Human Methylation450 K arrays. Methylation arrays are increasingly used in routine pathology diagnostics and can be applied to biopsy specimens, even on FFPE tissue. Our risk score is capable of confidently stratifying patients into *Low* and *High risk* of developing local recurrences, metastasis, and/or death. Therefore, CHROME has great potential to be integrated in the clinical management of chondrosarcoma patients.

## Author Contributions

**Sara Cardoso:** conceptualization, methodology, data curation, investigation, data analysis, data visualization, software development, writing – original draft, writing – review and editing. **Baptiste Ameline:** investigation, writing – review and editing. **Sanne Venneker:** investigation, writing – review and editing. **Debora M. Meijer:** investigation, writing – review and editing. **Zeynep B. Erdem:** investigation, writing – review and editing. **Dina Ruano:** investigation, writing – review and editing. **Brendy E. van den Akker:** investigation, writing – review and editing. **Anne‐Marie Cleton‐Jansen:** investigation, writing – review and editing. **Jon Brugger:** investigation, writing – review and editing. **Inge H. Briaire‐de Bruijn:** investigation, writing – review and editing. **Claire H. J. Scholte:** investigation, writing – review and editing. **Michiel A. J. van de Sande:** investigation, writing – review and editing. **Felix Haglund de Flon:** investigation, writing – review and editing. **Daniel Baumhoer:** conceptualization, methodology, supervision, writing – original draft, writing – review and editing. **Noel F. C. C. de Miranda:** conceptualization, methodology, supervision, writing – original draft, writing – review and editing. **Judith V. M. G. Bovée:** conceptualization, methodology, supervision, writing – original draft, writing – review and editing.

## Funding

This work was financially supported by the intramural Leiden Center for Computational Oncology (LCCO) strategic fund. Noel F.C.C. de Miranda is funded by the European Research Council (ERC) under the European Union's Horizon 2020 Research and Innovation Program (grant agreement no. 852832) and by the VIDI ZonMW (project number: 09150172110092). Sanne Venneker was financially supported by the BCRT grant 8922.

## Ethics Statement

Approval of institutional review board (Medisch‐Ethische Toetsingscommissie Leiden Den Haag Delft) was obtained for the use of tissue samples from the LUMC bone and soft tissue tumor biobank (Leiden, the Netherlands) (B20.067). Written informed consent from patients was obtained. All specimens were pseudoanonymized and handled according to the ethical guidelines described in ‘Code for Proper Secondary Use of Human Tissue in The Netherlands’ of the Dutch Federation of Medical Scientific Societies. The study of the Karolinska cohort was approved by Etikprövningsmyndigheten (reference 2022‐05409‐01).

## Conflicts of Interest

The authors declare no conflicts of interest.

## Supporting information


**Figure S1:** Discovery cohort. Kaplan–Meier survival estimate curves of disease‐free survival (DFS) for IDH mutation status without the ACT cases. *MUT*: IDH mutated; *WT*: IDH wild‐type. *Number at risk* table: number of cases through time with no event or not censored yet. *Cumulative number of events* table: cumulative number of cases through time with an event.
**Figure S2:** Discovery cohort. Kaplan–Meier survival estimate curves of disease‐free survival (DFS) for (A) anatomical location for all cases, (B) anatomical location without the ACT/G1 cases, and (C) TERT promoter mutational status. *MUT*: IDH mutated; *WT*: IDH wild‐type. *Number at risk* table: number of cases through time with no event or not censored yet. *Cumulative number of events* table: cumulative number of cases through time with an event.
**Figure S3:** Discovery dataset. Clustergram of the different clustering resolutions tested.
**Figure S4:** Visualization of discovery cohort's cases in the 2‐dimensional space using UMAP. Plots are colored according to (A) anatomical location, (B) maximum tumor size, in cm, and (C) TERT promoter mutational status. *MUT*: TERT promoter mutation present; *WT*: TERT promoter mutation absent.
**Figure S5:** Discovery cohort, only grades 2 and 3 cases. Kaplan–Meier survival estimate curves of disease‐free survival (DFS) for categorical CHROME risk. *Number at risk*: number of cases through time with no event and not censored yet. *Cumulative number of events*: cumulative number of cases through time with an event.
**Figure S6:** Representative H&E images of two high‐grade cases from the training dataset that were classified as *Low risk*. (A) Case L5037, H&E, ×10. Chondrogenic tumor with increased cellularity, cytologic atypia, and myxoid matrix change. Spindled chondrocytes are present at the periphery of the lobules. The histomorphological features are consistent with chondrosarcoma, grade 3. (B) Case L6648, H&E, ×8. Chondrogenic tumor with lobular architecture. The left side of the image shows clustered chondrocytes with mild‐to‐moderate cytologic atypia embedded in cartilaginous matrix. The right side shows increased cellularity, pronounced chondrocyte atypia, and myxoid matrix change. This case was diagnosed as chondrosarcoma, grade 2.
**Figure S7:** Within the discovery dataset, correlation between the probes from CHROME that were substituted and the new probes, chosen to make a 450 K‐compatible CHROME. (A) Probe cg06031622 was substituted by probe cg25336892, while (B) probe cg09678323 was substituted by probe cg10663897.
**Figure S8:** Correlation between the original risk score (CHROME) and the risk score adapted for 450 K Illumina arrays (450 K‐compatible CHROME) in the *Karolinska* dataset of the validation cohort.
**Figure S9:** Visualization of validation cohort's cases in the 2‐dimensional space using UMAP. Plots are colored according to (A) Tissue, (B) Dataset.
**Figure S10:** Visualization of validation cohort's cases in the 2‐dimensional space using UMAP. Plot colored according to anatomical location.
**Figure S11:** Validation cohort. Kaplan–Meier survival estimate curves of disease‐free survival (DFS) for anatomical location. *Number at risk*: number of cases through time with no event and not censored yet. *Cumulative number of events*: cumulative number of cases through time with an event.
**Figure S12:** Kaplan–Meier survival estimate curves of disease‐free survival (DFS) for the FFPE cases of the *Karolinska* dataset in the validation cohort.
**Figure S13:** Validation cohort, only grades 2 and 3 cases. Kaplan–Meier survival estimate curves of overall survival (OS) for categorical CHROME risk for (A) the full validation cohort, (B) only the Remy dataset and (C) only the Karolinska dataset. (D) Kaplan–Meier survival estimate curves of disease‐free survival (DFS) for Karolinska dataset. *Number at risk*: number of cases through time with no event and not censored yet. *Cumulative number of events*: cumulative number of cases through time with an event.

**Table S1:** Differentially methylated probes between clusters 1 + 2 and 3 + 4, and individual effect on survival of the differentially methylated probes.
**Table S2:** Follow‐up times in months of censored cases (cases without an event) with grades 2 or 3. The information is reported in the following manner: *n* = number of cases, mean (minimum to maximum).
**Table S3:** Effect of histological grade, IDH mutational status, age at diagnosis and sex with CHROME on overall survival (OS) for the full validation cohort and for the Remy and Karolinska datasets separately. Those with an individual effect were tested together in multivariate analysis. Results show the hazard ratios with respective *p* value. Grade, IDH status and sex were transformed into numerical values the following way: (1) ACT, (2) G2, (3) G3; (1) IDH wild‐type, (2) IDH mutated; (1) Female, (2) Male.

## Data Availability

The data generated for the discovery dataset is publicly available in Gene Expression Omnibus (GEO) at *GSE280135*. CHROME is available as an R package at https://git.lumc.nl/bstp/Tools/chrome. The code developed to produce CHROME is available at https://git.lumc.nl/bstp/Tools/chrome/tree/paper.
