# Supplementary material for: Accurate Prediction of Central Conventional Chondrosarcoma Risk and Outcome Using Methylation Profiling: CHROME
Source: Genes Chromosomes Cancer. 2026 Aug 4;65(8):e70160. doi: 10.1002/gcc.70160 (PMC13437929; doi:10.1002/gcc.70160)
Supplement: Supplementary file 1 [file GCC-65-e70160-s001.pdf]

Accurate prediction of central conventional CHondrosarcoma Risk and Outcome using MEthylation profiling: CHROME

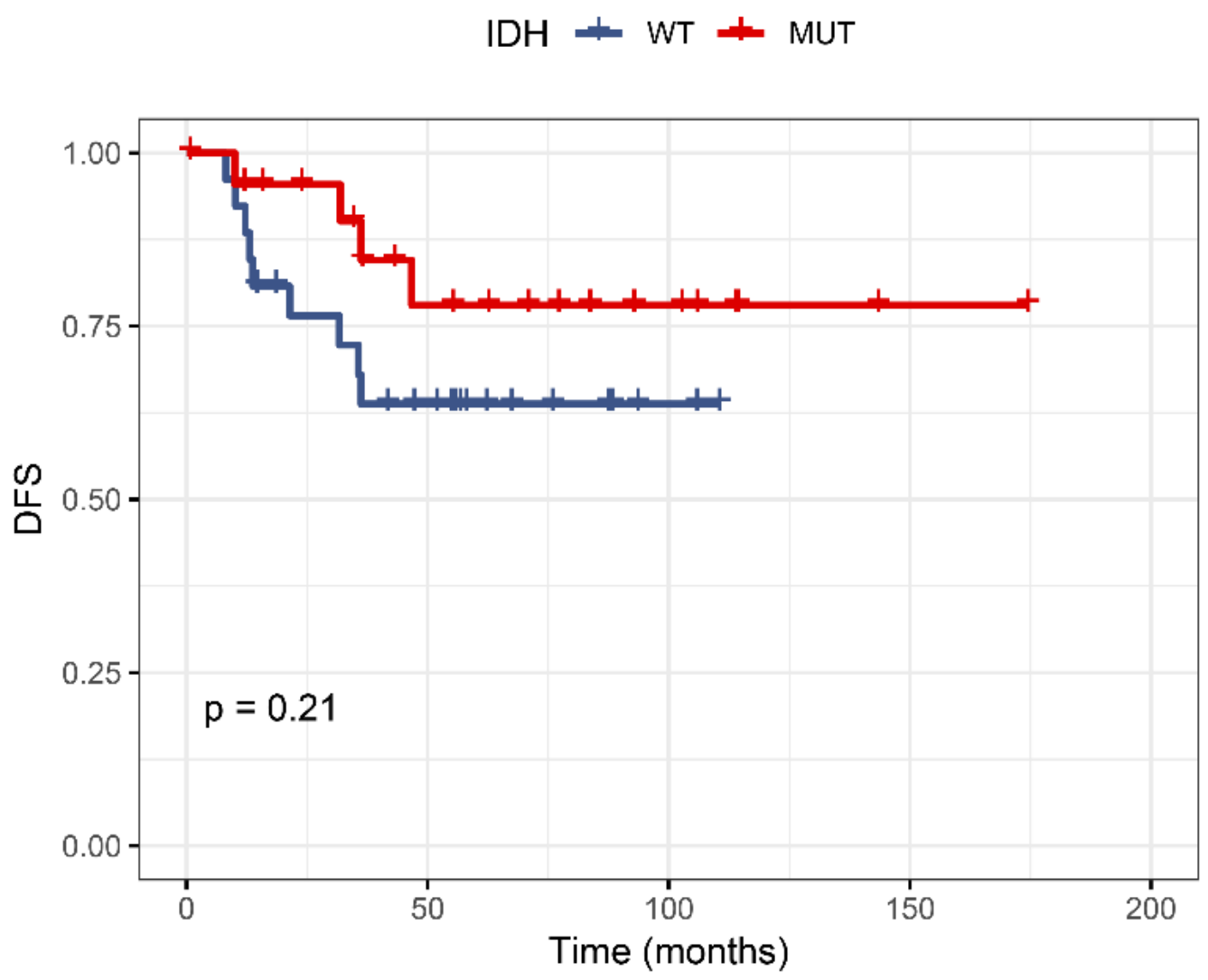

Number at risk

	0	50	100	150	200
WT	26	13	2	0	0
MUT	23	12	6	1	0

Cumulative number of events

	0	50	100	150	200
WT	0	9	9	9	9
MUT	0	4	4	4	4

Supplementary Figure S1: Discovery cohort. Kaplan-Meier Survival Estimate Curves of Disease-Free survival (DFS) for IDH mutation status without the ACT cases. *WT*: IDH wild-type; *MUT*: IDH mutated. *Number at risk* table: number of cases through time with no event or not censored yet. *Cumulative number of events* table: cumulative number of cases through time with an event.

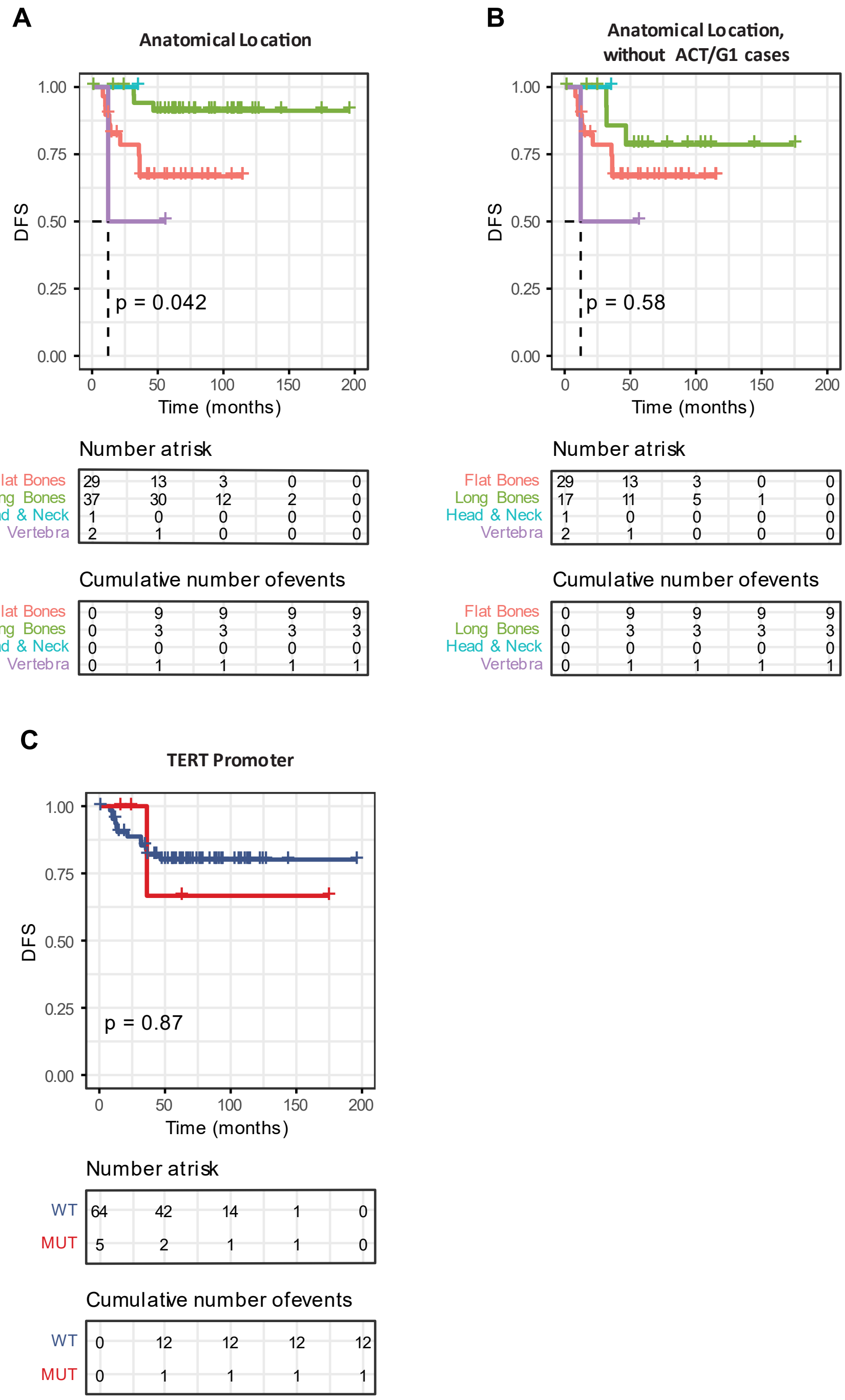

Supplementary Figure S2: Discovery cohort. Kaplan-Meier Survival Estimate Curves of Disease-Free survival (DFS) for (A) anatomical location for all cases, (B) anatomical location without the ACT/G1 cases, and (C) TERT promoter mutational status. *WT*: IDH wild-type; *MUT*: IDH mutated. *Number at risk* table: number of cases through time with no event or not censored yet. *Cumulative number of events* table: cumulative number of cases through time with an event.

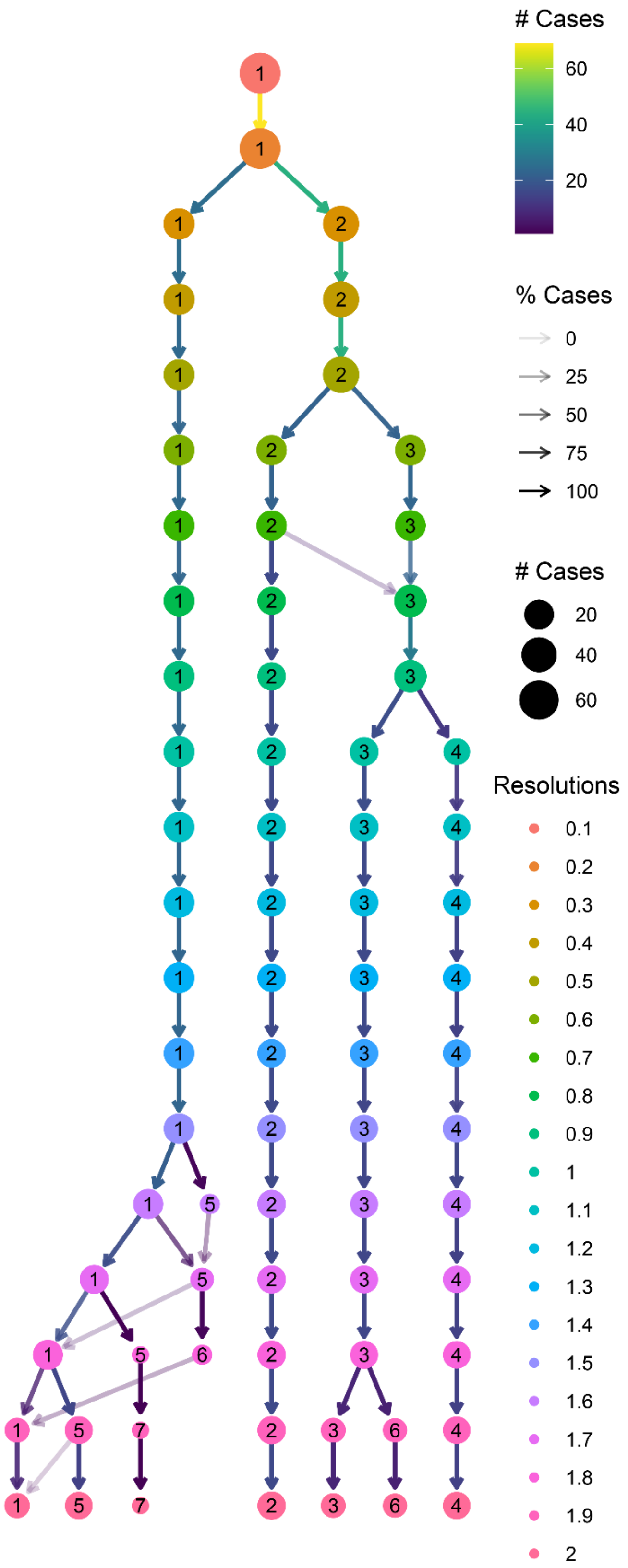

Supplementary figure S3: Discovery dataset. Clustergram of the different clustering resolutions tested.

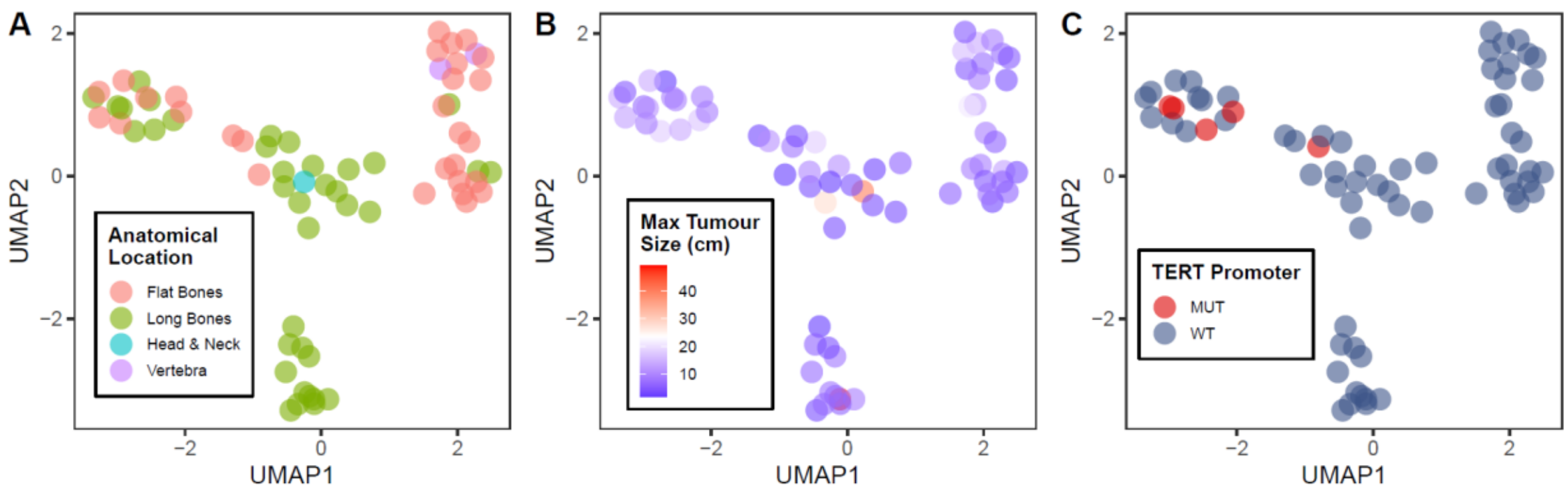

Supplementary Figure S4: Visualization of discovery cohort's cases in the 2-dimensional space using UMAP. Plots are coloured according to (A) anatomical location, (B) maximum tumour size, in cm, and (C) TERT promoter mutational status. *WT*: TERT promoter mutation absent; *MUT*: TERT promoter mutation present.

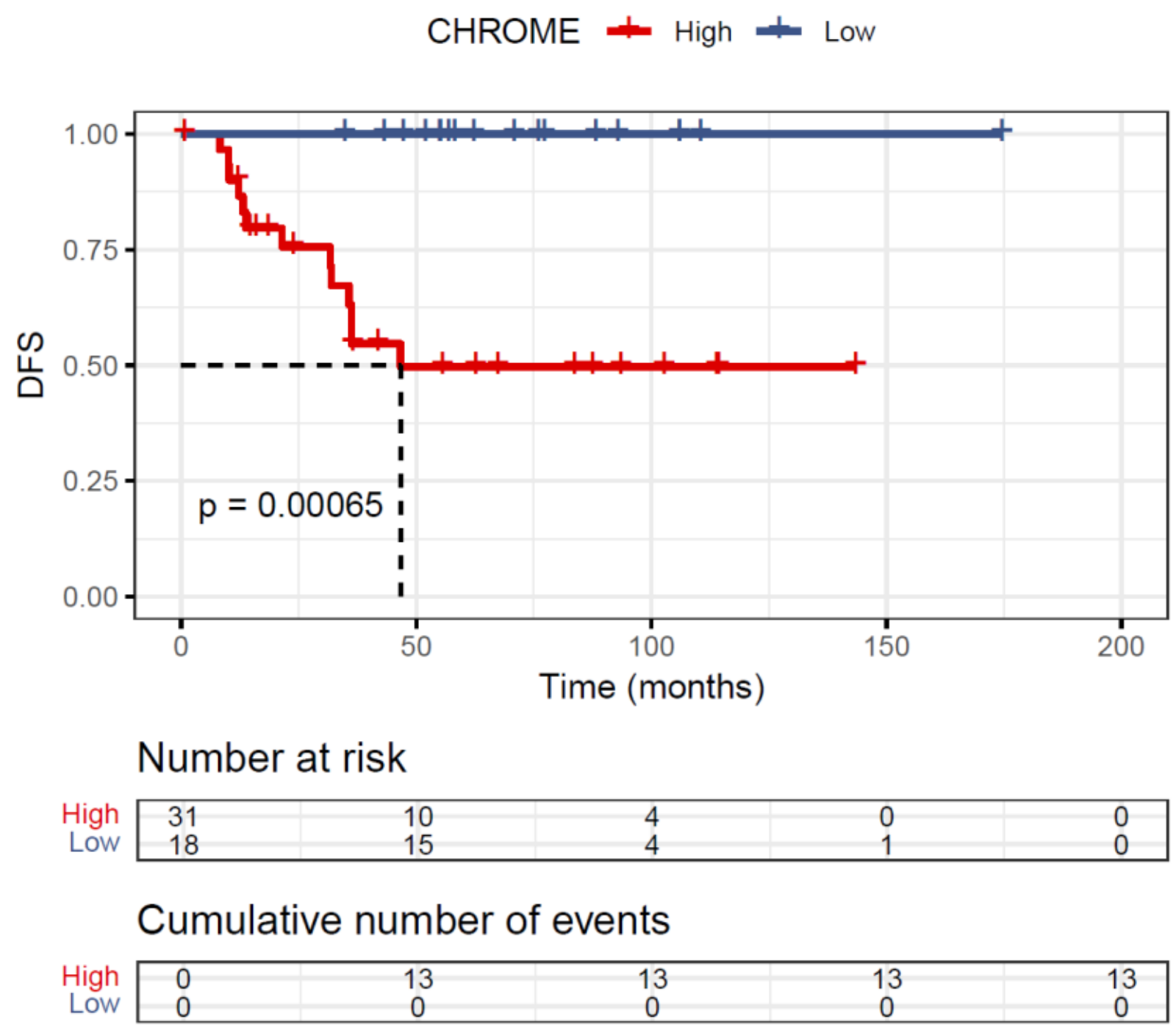

Supplementary Figure S5: Discovery cohort, only grades 2 and 3 cases. Kaplan-Meier Survival Estimate Curves of Disease-Free survival (DFS) for categorical CHROME risk. *Number at risk*: number of cases through time with no event and not censored yet. *Cumulative number of events*: cumulative number of cases through time with an event.

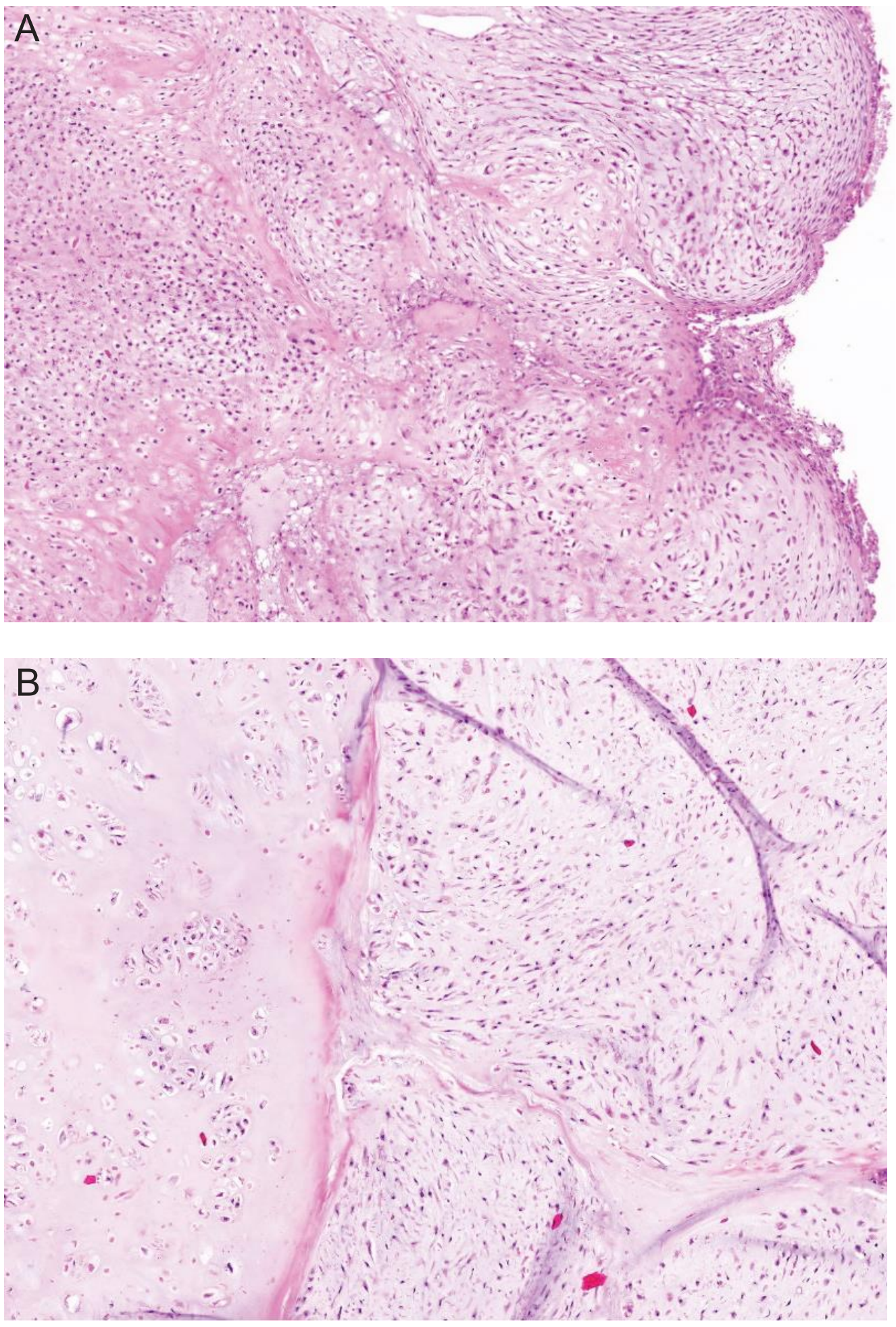

Supplementary figure S6: Representative H&E images of two high-grade cases from the training dataset that were classified as *Low risk*. (A) Case L5037, H&E, ×10. Chondrogenic tumour with increased cellularity, cytologic atypia, and myxoid matrix change. Spindled chondrocytes are present at the periphery of the lobules. The histomorphological features are consistent with chondrosarcoma, grade 3. (B) Case L6648, H&E, ×8. Chondrogenic tumour with lobular architecture. The left side of the image shows clustered chondrocytes with mild-to-moderate cytologic atypia embedded in cartilaginous matrix. The right side shows increased cellularity, pronounced chondrocyte atypia, and myxoid matrix change. This case was diagnosed as chondrosarcoma, grade 2.

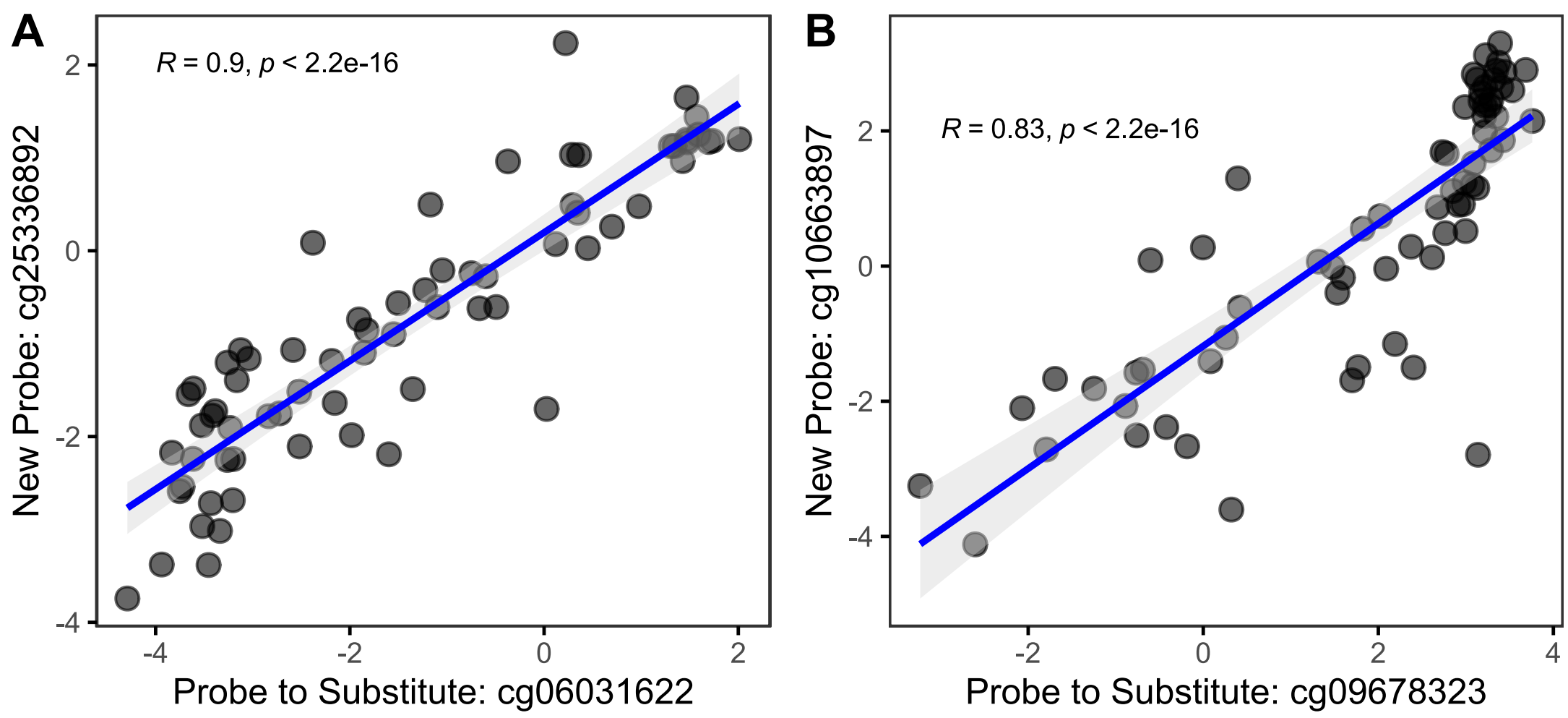

Supplementary Figure S7: Within the discovery dataset, correlation between the probes from CHROME that were substituted and the new probes, chosen to make a 450K-compatible CHROME. (A) Probe cg06031622 was substituted by probe cg25336892, while (B) probe cg09678323 was substituted by probe cg10663897.

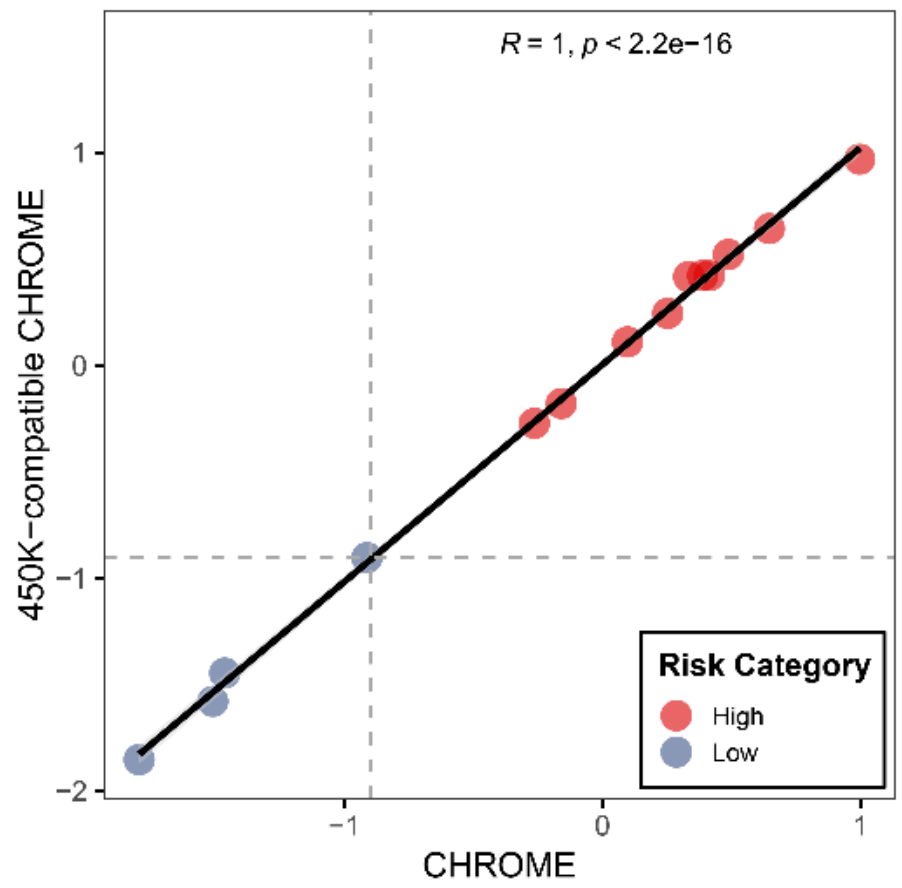

Supplementary Figure S8: Correlation between the original risk score (CHROME) and the risk score adapted for 450K Illumina arrays (450K-compatible CHROME) in the *Karolinska* dataset of the validation cohort.

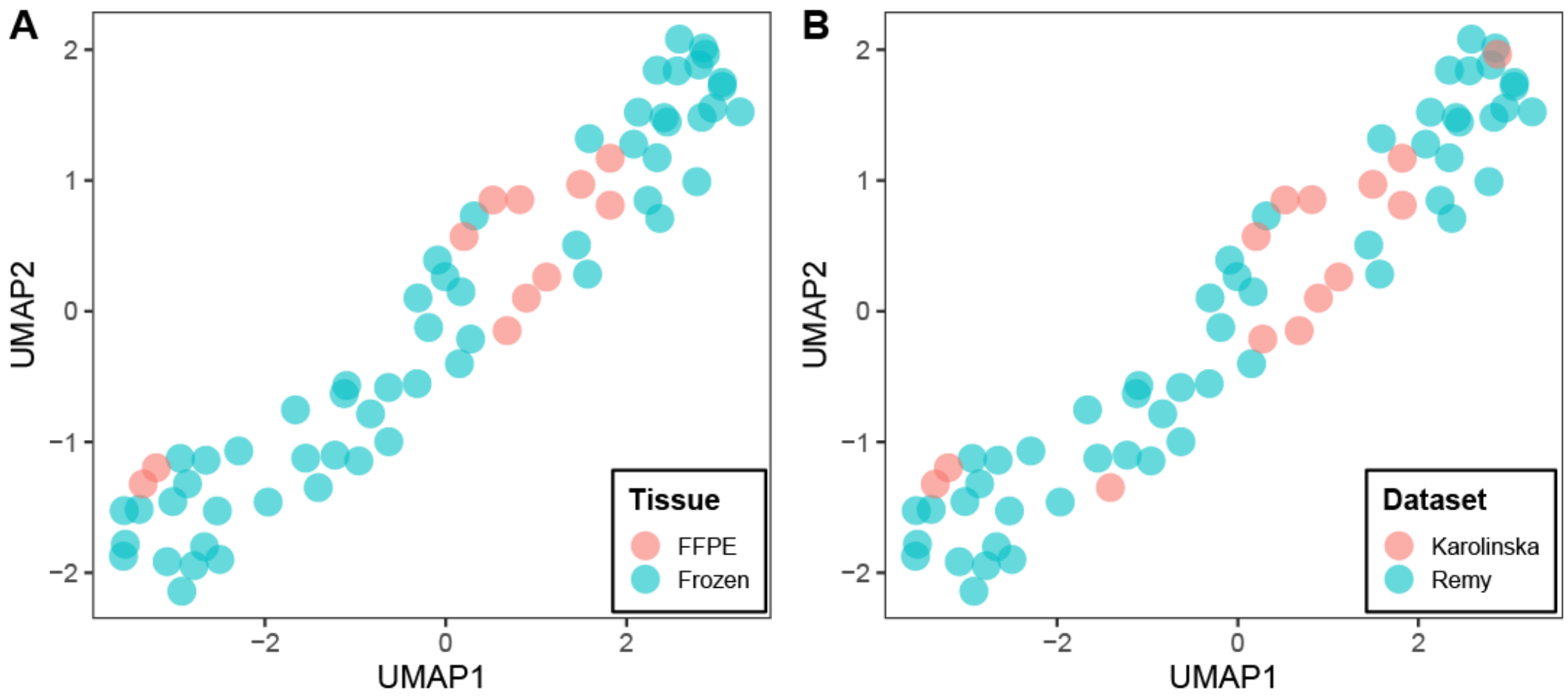

Supplementary Figure S9: Visualization of validation cohort's cases in the 2-dimensional space using UMAP. Plots are coloured according to (A) Tissue, (B) Dataset.

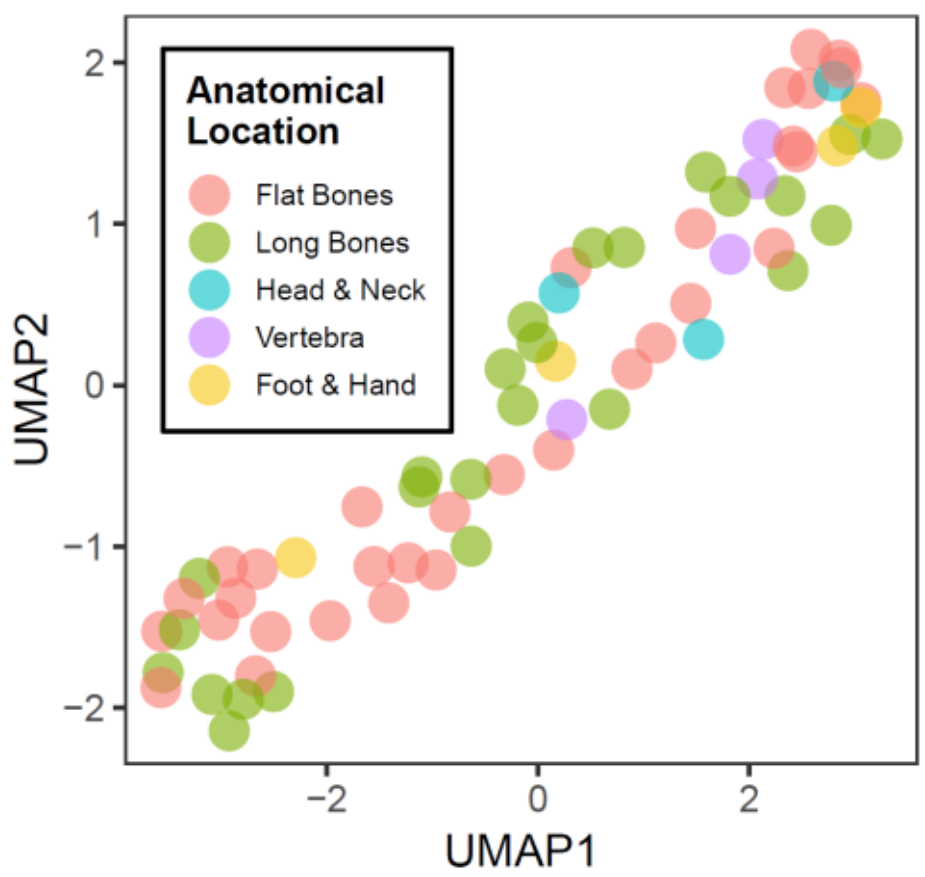

Supplementary Figure S10: Visualization of validation cohort's cases in the 2-dimensional space using UMAP. Plot coloured according to anatomical location.

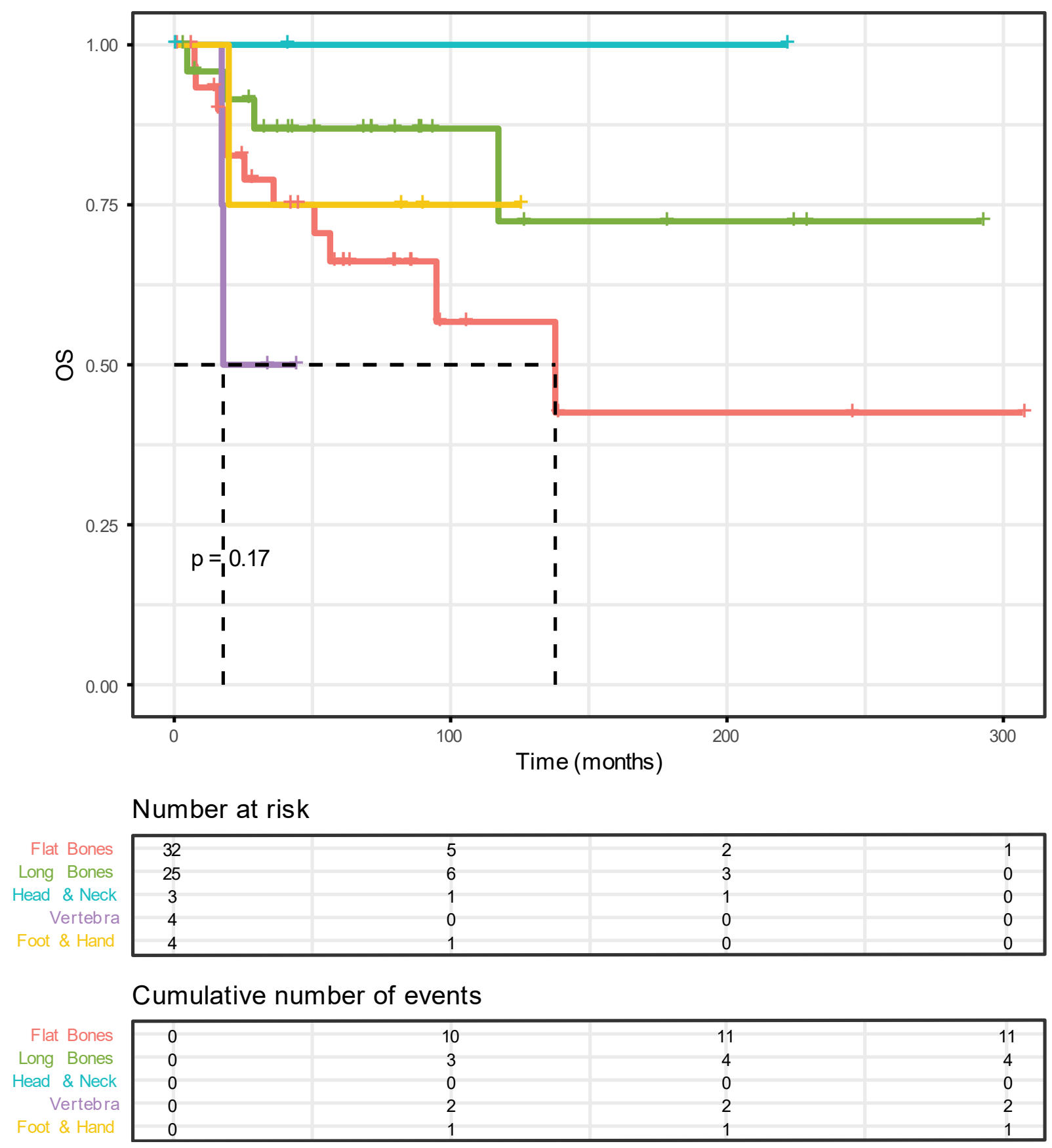

Supplementary Figure S11: Validation cohort. Kaplan-Meier survival estimate curves of disease-free survival (DFS) for anatomical location. *Number at risk*: number of cases through time with no event and not censored yet. *Cumulative number of events*: cumulative number of cases through time with an event.

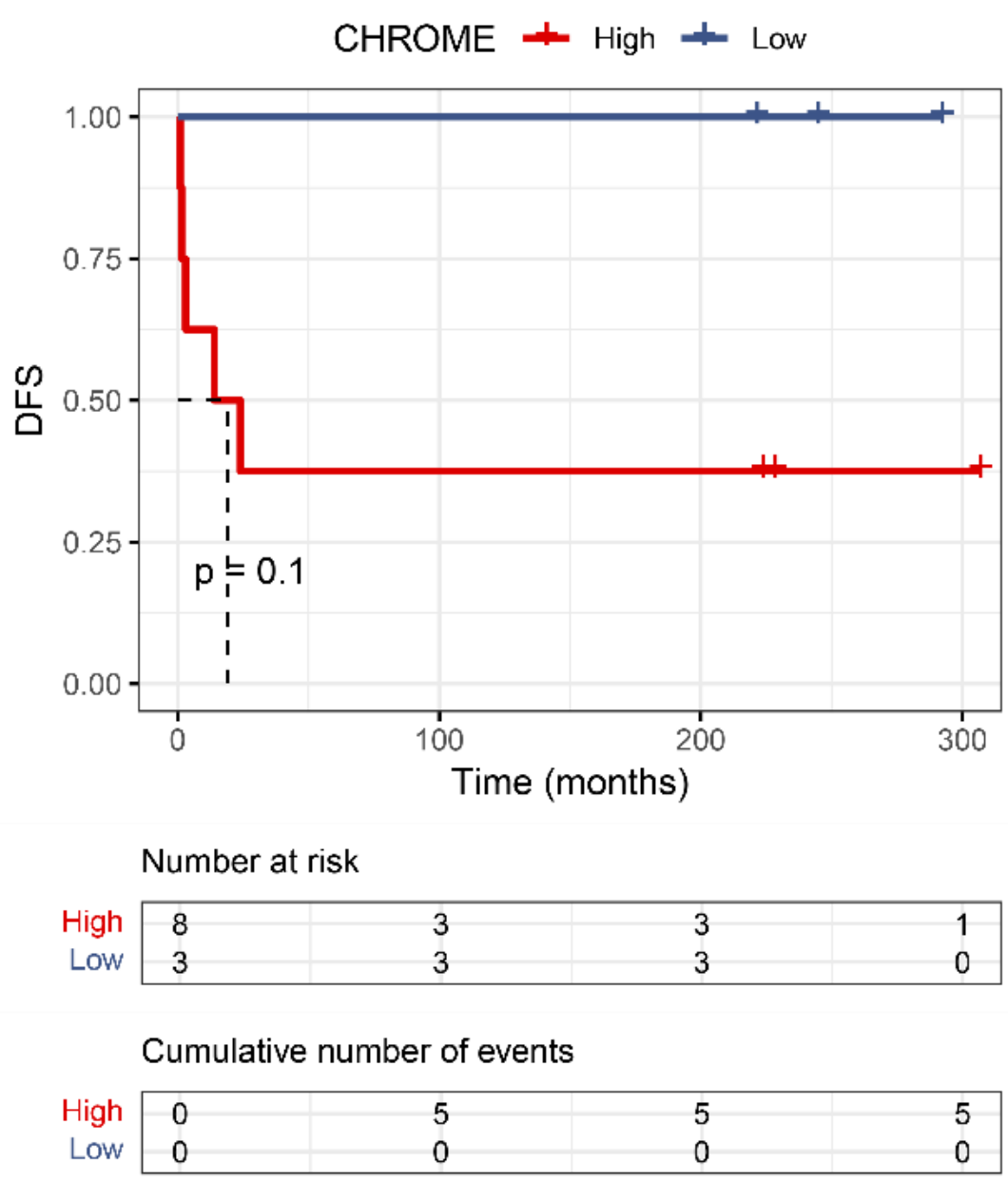

Supplementary Figure S12: Kaplan-Meier survival estimate curves of disease-free survival (DFS) for the FFPE cases of the *Karolinska* dataset in the validation cohort.

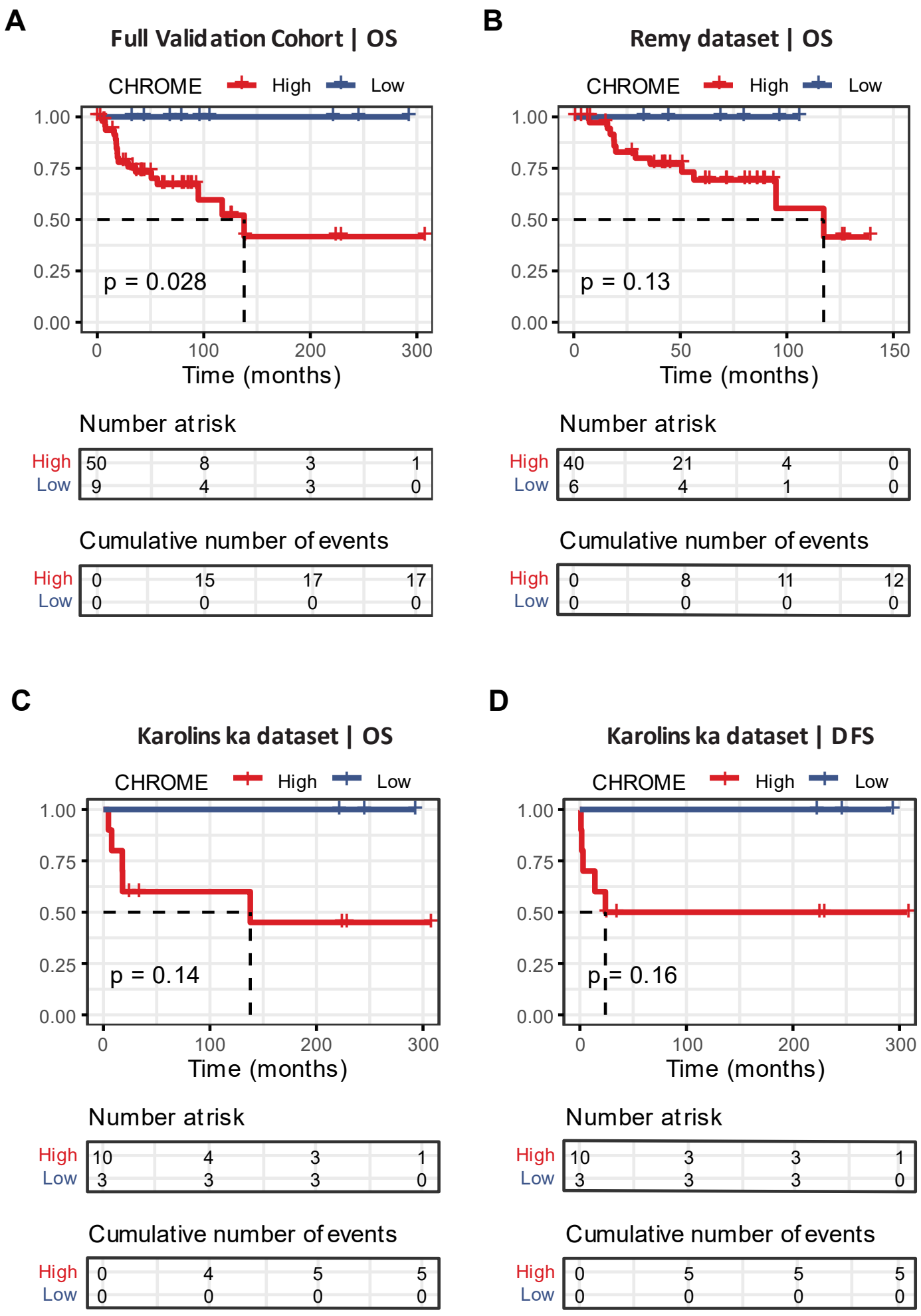

Supplementary figure S13: Validation cohort, only grades 2 and 3 cases. Kaplan-Meier survival estimate curves of overall survival (OS) for categorical CHROME risk for (A) the full validation cohort, (B) only the Remy dataset and (C) only the Karolinska dataset. (D) Kaplan-Meier survival estimate curves of disease-free survival (DFS) for Karolinska dataset. *Number at risk*: number of cases through time with no event and not censored yet. *Cumulative number of events*: cumulative number of cases through time with an event.
